# GLP-1 Receptor Agonists in Heart Failure

**DOI:** 10.3390/biom15101403

**Published:** 2025-10-02

**Authors:** Ali Reza Rahmani, Simrat Kaur Dhaliwal, Paola Pastena, Eliot Kazakov, Keerthana Jayaseelan, Andreas Kalogeropoulos

**Affiliations:** 1 Division of Cardiology, Department of Medicine, Stony Brook University, Stony Brook, NY 11790, USA; alireza.rahmani@stonybrookmedicine.edu (A.R.R.);; 2 Division of Cardiology, Department of Pediatrics, Columbia University Irving Medical Center, New York, NY 10032, USA; 3 Renaissance School of Medicine, Stony Brook University, Stony Brook, NY 11790, USA; 4 Department of Medicine, Stony Brook University, Stony Brook, NY 11794, USA; 5 Health Sciences Center, Stony Brook University Medical Center, Stony Brook, NY 11794, USA

**Keywords:** heart failure, GLP-1 RAs, molecular pathways, pathophysiology

## Abstract

Heart failure (HF) is a growing public health concern, driven by the increasing prevalence of obesity, diabetes, and aging. Despite therapeutic advances, HF continues to be associated with high morbidity and mortality. Glucagon-like peptide-1 receptor agonists (GLP-1 RAs), originally developed for glycemic control in type 2 diabetes, have demonstrated cardiovascular benefits in clinical trials. Recent studies, including STEP-HFpEF and SUMMIT, have shown improvement in symptoms and weight loss in patients with HF with preserved ejection fraction (HFpEF). GLP-1 RAs are involved in multiple biological pathways relevant to heart failure pathophysiology. These include pathways related to sympathetic nervous system activity, inflammatory cytokine signaling, oxidative stress, calcium handling, natriuretic peptide signaling, and cardiac metabolism. GLP-1 receptor agonists modulate vascular pathways involving nitric oxide signaling, endothelial function, and renal sodium handling, contributing to improved hemodynamics and neurohormonal balance. Together, these actions intersect with key neurohormonal and cellular processes contributing to chronic heart failure progression. This review explores the mechanistic overlap between GLP-1 receptor signaling and heart failure pathophysiology. This mechanistic overlap suggests a plausible role for these agents as adjunctive treatments in heart failure, especially in metabolically driven phenotypes. While direct cardiac effects remain incompletely defined, systemic metabolic and anti-inflammatory actions provide a mechanistic basis for observed clinical benefits.

## 1. Introduction

Approximately 6.7 million Americans over the age of 20 suffer from HF (HF), and the prevalence is projected to nearly double by 2050 [1]. The European Society of Cardiology defines HF as a clinical syndrome resulting from structural and/or functional cardiac abnormalities, accompanied by signs or symptoms of pulmonary or systemic congestion and/or elevated levels of natriuretic peptides [2]. Numerous risk factors have been implicated in the development of HF, with obesity, recognized as a body mass index (BMI ≥ 30 kg/m^2^), being one of the most significant contributors [3]. The Framingham Heart Study was instrumental in evaluating the impact of obesity on HF. Involving 5,881 participants, the study demonstrated that for each 1-unit increase in BMI, the incidence of HF rose by 5% in men and 7% in women [4]. These findings have been successfully replicated in future studies [4,5]. Additional studies suggest that elevated BMI is more strongly associated with HF with preserved ejection fraction (HFpEF) than with HF with reduced ejection fraction (HFrEF) [6,7]. However, irrespective of the HF subtype, patients with concurrent obesity are more likely to experience adverse hemodynamic profiles, more severe clinical manifestations, and overall reduced quality of life [8,9]. In light of the interrelated risks of obesity and HF, significant effort has been devoted to exploring treatment approaches, particularly those emphasizing lifestyle modifications and pharmacotherapy.

The potential of pharmacological therapies to alleviate HF symptoms and enhance functional capacity has been extensively investigated. Of the medications studied, glucagon-like peptide 1 receptor agonist (GLP-1 RA) have revolutionized the field of HF. This class of medication was initially utilized to treat type 2 diabetes mellitus. However, over the past several years, it gained a strong reputation for obesity management. This prompted consideration of whether the use of GLP-1 RAs should extend beyond the diabetic population. In a landmark study, Lincoff et al. demonstrated that weekly subcutaneous administration of GLP-1 RA was superior to placebo in reducing cardiovascular mortality, nonfatal myocardial infarction, and nonfatal stroke in patients with preexisting cardiovascular disease or overweight/obesity without diabetes [10]. Subsequently, the American Diabetes Association recognized GLP-1 RA as a first-line therapy for patients with either established or elevated risk of developing atherosclerotic cardiovascular disease [11]. Given their favorable effects on both mitigating adverse cardiovascular events and overall cardiometabolic risk factors, subsequent studies began to investigate the role of GLP-1 RAs in the context of HF. However, early investigations yielded inconclusive results. For instance, the FIGHT [12] trial, which studied the effects of liraglutide in patients recently hospitalized for acute decompensated heart failure, found no significant improvement in the primary endpoint compared to placebo. Similarly, the LIVE [13] trial also showed that Liraglutide did not affect left ventricular systolic function compared with placebo in stable chronic heart failure patients with and without diabetes.

One of the recent studies investigating the role of GLP-1 RA in HF was the STEP-HFpEF Trial in which 529 HFpEF patients with obesity were randomly assigned to receive either a GLP-1 RA (semaglutide) or placebo for 52 weeks [14]. Of note, patients with diabetes were strictly excluded from the study. Patients treated with semaglutide experienced larger reductions in HF symptoms and physical limitations, greater improvements in exercise function, and greater weight loss when compared to the placebo group. A second pivotal trial was published, evaluating the effects of GLP-1 RAs in patients with HFpEF and coexisting type 2 diabetes [15]. Similarly, the use of GLP-1 RA (when compared to the placebo group) led to larger reductions in HF symptoms and physical limitations, as well as greater weight loss. In contrast, the data on the use of GLP-1RAs in patients with HFrEF remains limited and inconclusive, highlighting the need for further investigation [16].

Despite multiple studies highlighting the potential benefits of GLP-1 RAs in patients with HF, their mechanism of action in HF remains elusive. The underlying uncertainty is attributable to several factors, notably the low expression of GLP-1 receptors in adult human cardiomyocytes, which limits the ability to investigate direct myocardial effects [17]. Additionally, the majority of observed cardiovascular benefits appear to be indirect, primarily mediated through weight reduction, improved glycemic control, and systemic metabolic improvements. Moreover, various trials, including STEP-HFpEF, have demonstrated benefits of GLP-1 RAs in non-diabetic patients, suggesting the involvement of mechanisms beyond glycemic control [14]. In this paper, we present a comprehensive review of the evidence linking GLP-1 RA signaling pathways to the neurohormonal mechanisms underlying HF, with the aim of enhancing our understanding of the physiological effects of GLP-1 RAs. The complex mechanisms investigated include the systemic nervous system activation, renin–angiotensin–aldosterone system, inflammation/oxidative stress, calcium handling and natriuretic peptide system.

## 2. GLP-1 Receptor Agonists Biology in Heart Failure

Glucagon-like peptide-1 receptor agonists are synthetic peptides that bind to and activate the GLP-1 receptor, a class B G protein–coupled receptor [18]. The GLP-1R is most abundantly expressed on pancreatic β-cells, where it mediates glucose-dependent insulin secretion, but is also present in the central nervous system, gastrointestinal tract, kidneys, lungs, and at lower density in cardiovascular tissues including cardiomyocytes, endothelial cells, and vascular smooth muscle cells [19]. The distribution of GLP-1 receptors within the human heart remains incompletely defined, with immunohistochemical studies reporting receptor positive cells primarily in the sinoatrial node and atrial tissue, though findings across cardiac regions are inconsistent [17,20]. Complementary functional evidence, however, supports cardiomyocyte GLP-1R activity, as semaglutide was shown to attenuate diabetic cardiac inflammation through a Sirt3–RKIP–NF-κB pathway in rodent cardiomyocytes [21]. In another study, semaglutide normalized abnormal calcium transients in cardiomyocytes from obese rats, restoring calcium handling toward physiological levels and improving excitation–contraction coupling [22]. Overall, although receptor density in the heart is low compared with pancreatic tissue, early preclinical evidence suggests potential GLP-1R activity in cardiomyocytes. These findings support the presence of GLP-1R activity in heart muscle cells; however, limitations in current detection techniques, variability between assays, and low receptor abundance continue to make precise localization of cardiac GLP-1R challenging. Therefore, clinical relevance remains to be established.

**Figure 1:**
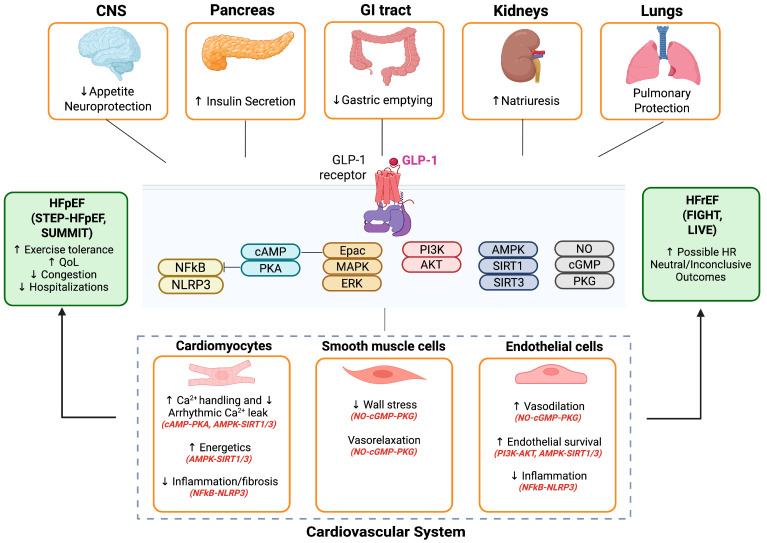
Modulatory Role of GLP-1 Receptor Agonists in Cardiomyocytes and Heart Failure. AMPK—AMP-activated protein kinase; Akt—Protein kinase B; cAMP—Cyclic adenosine monophosphate; cGMP—Cyclic guanosine monophosphate; CNS—Central nervous system; Epac—Exchange protein directly activated by cAMP; ERK—Extracellular signal-regulated kinase; GI tract—Gastrointestinal tract; GLP-1—Glucagon-like peptide-1; HFpEF—Heart failure with preserved ejection fraction; HFrEF—Heart failure with reduced ejection fraction; HR—Hazard ratio; MAPK—Mitogen-activated protein kinase; NFκB—Nuclear factor kappa B; NLRP3—NOD-like receptor protein 3; NO—Nitric oxide; PKA—Protein kinase A; PKG—Protein kinase G; PI3K—Phosphoinositide 3-kinase; QoL—Quality of life; SIRT1/3—Sirtuin 1/3. Figure created with in BioRender. Pastena, P. (2025) https://BioRender.com/akngcnd( accessed 19 September 2025).

In cardiomyocytes when GLP-1 receptor agonists bind to the GLP-1 receptor, they activate a broad network of intracellular signaling pathways [23]. The central event is the cAMP–PKA axis, from which multiple downstream cascades arise [18]. These include the PI3K/Akt pathway, the MAPK/ERK1/2 pathway, and the PKG–PKCε axis, which are involved in survival and stress-adaptation signaling. In addition, GLP-1R stimulation engages the AMPK–sirtuin pathway, regulating mitochondrial metabolism and oxidative balance [24]. Finally, GLP-1RAs influence inflammatory and fibrotic signaling through inhibition of NF-κB, suppression of the NLRP3 inflammasome, and attenuation of TGF-β/Smad signaling [23]. Importantly, these signaling events are not limited to the myocardium. The cAMP–PKA axis, AMPK–sirtuin networks, and anti-inflammatory pathways activated by GLP-1RAs operate across multiple organ systems, including the vasculature, kidneys, adipose tissue, and central nervous system [19,24]. Thus, the cardiomyocyte responses described likely reflect a broader integrative biology in which GLP-1R signaling coordinates metabolic adaptation, oxidative balance, immune modulation, and fibrosis suppression throughout the body. The widespread expression of GLP-1 receptors underlies the ability of GLP-1RAs to influence pathways beyond glucose metabolism, promoting cardiovascular, renal, and metabolic protection that together contribute to improved outcomes in HF patients specifically HFpEF. In addition to effects in the myocardium, vasculature, and central nervous system, GLP-1 receptor agonists also act within the kidney [19,25]. GLP-1 receptors are expressed in proximal tubular epithelial cells, where their activation promotes natriuresis and diuresis through inhibition of the sodium–hydrogen exchanger 3 (NHE3) [25]. These actions reduce sodium reabsorption and intravascular volume, thereby indirectly modulating RAAS activity and contributing to blood pressure and afterload reduction [26] (Figure 1).

In recent preclinical studies, semaglutide favorably modulates cardiac remodeling in a pressure overload-induced heart failure model [27]. In mice subjected to transverse aortic constriction, semaglutide treatment attenuated left ventricular hypertrophy, reduced interstitial fibrosis, and improved systolic and diastolic function. Tian et al. [28] demonstrated that semaglutide improved cardiac function by enhancing mitochondrial respiration and calcium handling, preserving mitochondrial structure, and promoting mitochondrial quality control. These effects were accompanied by activation of AMPK, which contributed to maintaining cardiomyocyte integrity and overall myocardial performance. In another study, Li et al. [29] demonstrated that semaglutide attenuates excessive exercise-induced myocardial injury in rats by inhibiting oxidative stress and suppressing inflammatory signaling. Specifically, semaglutide reduced ROS generation and downregulated NF-κB activation along with pro-inflammatory cytokines such as TNF-α and IL-1β, while also limiting apoptosis in cardiomyocytes. Similarly, liraglutide has been shown to protect cardiomyocytes from IL-1β–induced metabolic disturbance and mitochondrial dysfunction. This protection was associated with preserved mitochondrial membrane potential, reduced ROS production, suppression of NF-κB signaling, and activation of AMPK, thereby maintaining cellular energy homeostasis and mitigating inflammation-driven injury [30]. Krammer et al. [31] further extended these findings by demonstrating that semaglutide directly enhances contractility in isolated human cardiomyocytes. Semaglutide exerted cardioprotective actions by improving contractile performance and attenuating stress-induced injury in human ventricular myocardium via inhibition of late sodium current and reduction in sarcoplasmic reticulum calcium leaks. In myocardial infarction models [32,33,34], semaglutide improved ventricular function, reduced fibrosis, and corrected metabolic disturbances, while in ischemia–reperfusion injury it decreased infarct size and preserved mitochondrial integrity, findings that align with core mechanisms driving heart failure progression.

Clinical evidence has provided mixed results regarding the role of GLP-1RAs in heart failure. In HFrEF, both the FIGHT [12] and LIVE [13] trials with liraglutide were neutral, showing no improvements in mortality, hospitalizations, ventricular function, or biomarkers, with LIVE additionally reporting increased heart rate and more cardiac adverse events in a small, heterogeneous population. By contrast, in HFpEF, GLP-1 RAs have shown consistent benefit. STEP-HFpEF [14] demonstrated that semaglutide improved symptoms, exercise capacity, NT-proBNP, and body weight in obese patients, while SUMMIT [35] further extended these findings by showing that tirzepatide not only improved symptoms and biomarkers but also reduced HF hospitalizations and composite clinical outcomes. Clinical findings partly reflect preclinical observations that GLP-1RAs can modulate pathways involving remodeling, calcium dynamics, mitochondrial stability, and inflammation, suggesting these effects could be particularly relevant in HFpEF rather than HFrEF (Table 1).

However, GLP-1RAs are generally well tolerated, several safety issues are relevant in HF. A recent meta-analysis in non-diabetic patients with overweight or obesity confirmed higher risks of nausea, vomiting, diarrhea, and constipation, particularly with semaglutide, liraglutide, and tirzepatide [36]. Although not conducted in HF, these findings mirror the adverse event patterns reported in HF trials. Across HF trials, gastrointestinal intolerance is the most consistent adverse effect of GLP-1RAs. In STEP-HFpEF [14], discontinuations due to adverse events were more common with semaglutide than placebo (35 vs. 14). In SUMMIT [35], tirzepatide was associated with higher rates of GI adverse events compared with placebo, including diarrhea (18.4% vs. 6.3%), nausea (17.0% vs. 6.5%), constipation (14.8% vs. 6.0%), and vomiting (10.4% vs. 2.2%). These effects contributed to treatment discontinuation in 6.3% of tirzepatide patients vs. 1.4% with placebo, underscoring GI intolerance as the principal limiting factor for long-term therapy. Importantly, despite these tolerability concerns, serious adverse events were not increased with GLP-1RAs. In the LIVE trial [13], gastrointestinal adverse events were again most prominent, with nausea (39 vs. 4 cases) and constipation (13 vs. 2) being more common with liraglutide than placebo. Dizziness (19 vs. 7) and fatigue (9 vs. 4) were the most frequent CNS-related events, while cardiac adverse events (13 vs. 10) were numerically higher with liraglutide, although worsening heart failure occurred more often with placebo (4 vs. 0). These findings highlight that gastrointestinal intolerance remains the main limiting factor for GLP-1RA therapy in HF, while cardiac and CNS events appear less consistent across trials.

## 3. Sympathetic Nervous System Activation in Heart Failure

**Figure 2:**
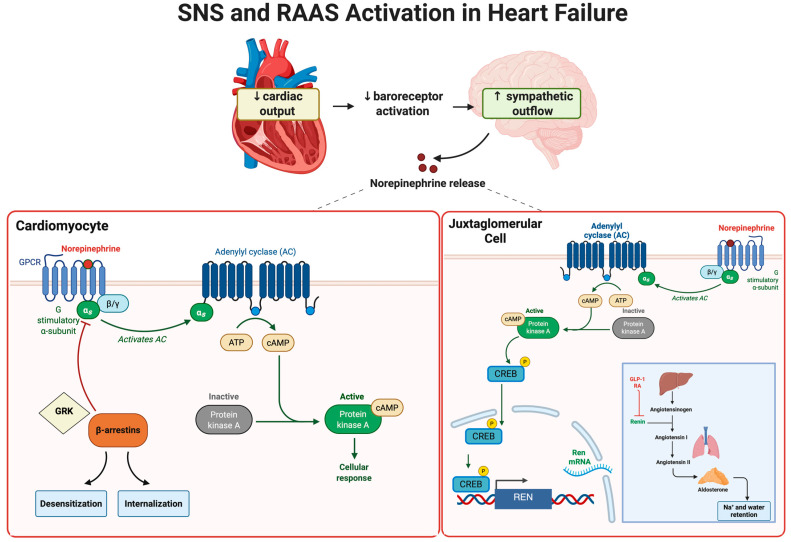
Sympathetic Nervous System and Renin–Angiotensin–Aldosterone System Activation in Heart Failure and Modulatory Role of GLP-1 Receptor Agonists. Although GLP-1 receptor agonists do not directly block renin but may modulate its expression through upstream signaling pathways. AC—adenylyl cyclase; ATP—Adenosine Triphosphate; cAMP—Cyclic Adenosine Monophosphate; CREB—cAMP Response Element-Binding Protein; GLP-1 RA—Glucagon-Like Peptide-1 Receptor Agonist; GPCR—G Protein-Coupled Receptor; GRK—G Protein-Coupled Receptor Kinase; mRNA—Messenger Ribonucleic Acid; PKA—Protein Kinase A; RAAS—Renin–Angiotensin–Aldosterone System; REN—Renin; SNS—Sympathetic Nervous System. Figure created with https://BioRender.com (accessed 4 August 2025).

**Figure 3:**
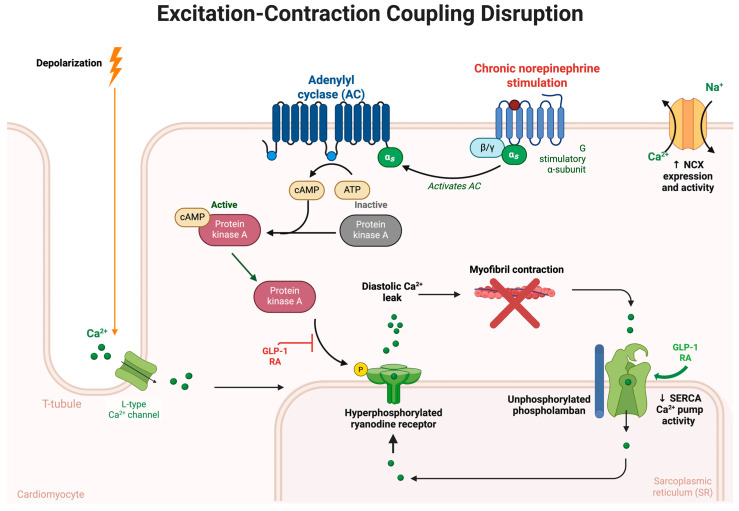
Excitation–Contraction Coupling Disruption in Heart Failure and Modulatory Role of GLP-1 Receptor Agonists. AC—Adenylyl Cyclase; ATP—Adenosine Triphosphate; cAMP—Cyclic Adenosine Monophosphate; Ca^2+^—Calcium Ion; GLP-1 RA—Glucagon-Like Peptide-1 Receptor Agonist; NCX—Sodium–Calcium Exchanger; PKA—Protein Kinase A; SR—Sarcoplasmic Reticulum. Figure created with https://BioRender.com (accessed 4 August 2025).

Heart failure is characterized by a reduction in cardiac output, triggering compensatory mechanisms intended to preserve perfusion and maintain hemodynamic stability. One critical response involves activation of the sympathetic nervous system (SNS). Although initially beneficial, prolonged SNS activation contributes significantly to disease progression, cardiac dysfunction, and poor clinical outcomes [37,38,39,40]. Under physiological conditions, cardiac output is modulated by a balance between sympathetic and parasympathetic tone. In HF, reduced cardiac output diminishes baroreceptor activity, triggering increased sympathetic outflow from the central nervous system [41]. This heightened sympathetic activation leads to excessive norepinephrine (NE) release, primarily acting on cardiac β1-adrenergic receptors to activate Gs protein signaling, stimulate adenylyl cyclase, and elevate intracellular cyclic adenosine monophosphate (cAMP) [39]. The resultant activation of protein kinase A (PKA) enhances calcium entry through L-type calcium channels (LTCCs), and facilitates sarcoplasmic reticulum (SR) calcium reuptake via phospholamban (PLB) phosphorylation and Sarcoplasmic/endoplasmic Ca^2+^-ATPase (SERCA2a) activation, thereby improving inotropy and enhanced diastolic relaxation (positive lusitropy) [42,43]. (Figure 1, Figure 2 and Figure 3).

While initially compensatory, chronic adrenergic stimulation induces β1-receptor desensitization and downregulation through G protein-coupled receptor kinases (GRKs) and β-arrestins, impairing contractile responsiveness and promoting intracellular calcium overload, oxidative stress, and arrhythmogenesis [43,44,45]. Additionally, sustained SNS activation drives peripheral vasoconstriction via α1-receptors and stimulates renin release through renal β1-receptors, activating the Renin–Angiotensin–Aldosterone System (RAAS) axis, increasing afterload and preload, and exacerbating congestion and remodeling [46,47,48]. Clinically, chronic sympathetic overactivity contributes to tachycardia, arrhythmias, fluid retention, and catabolic complications including cachexia and insulin resistance, correlating with worse prognosis in HF patients [49,50,51].

The cardioprotective effects of GLP-1 RAs are partly mediated through the elevation of intracellular cAMP, a second messenger integral to excitation–contraction coupling in cardiomyocytes [52]. Upon GLP-1 receptor activation, which is coupled to Gs proteins, adenylyl cyclase is stimulated, resulting in increased cAMP production [18,19]. Elevated cAMP levels activate PKA, which subsequently phosphorylates key regulatory proteins involved in calcium handling and contractility. Specifically, PKA phosphorylates LTCCs, enhancing calcium influx during the action potential [53] and ryanodine receptor 2 (RyR2), promoting calcium-induced calcium release from the SR [54,55]. In parallel, PKA-mediated phosphorylation of PLB alleviates its inhibitory effect on the SERCA2a, thereby accelerating calcium reuptake into the SR during diastole [56] (Figure 1, Figure 2 and Figure 3). Together, these actions lead to positive inotropy and positive lusitropy, restoring more efficient cardiac output [57,58]. While this intracellular cascade overlaps with β-adrenergic signaling induced by sympathetic nervous system activation, GLP-1-mediated cAMP elevation appears to confer similar functional benefits with reduced risk of maladaptive remodeling, arrhythmogenesis, or receptor desensitization [18,19,59,60]. In cardiomyocytes, β1-adrenergic stimulation drives sustained cAMP accumulation at the plasma membrane, leading to calcium overload, pro-apoptotic signaling, and progressive desensitization of β1-receptors during chronic heart failure [39]. By contrast, GLP-1R activation appears to generate more spatially and temporally regulated cAMP signals, with evidence for signaling at endosomal and mitochondrial compartments that support metabolic function and mitochondrial homeostasis rather than excessive contractile stimulation [19]. These differences may help explain why GLP-1R stimulation can provide some inotropic support without the same degree of maladaptive remodeling or arrhythmic risk typically associated with chronic β1-adrenergic activation. In experimental models [61], GLP-1 receptor activation has been linked to modest increases in intracellular cAMP. Such effects could potentially reduce reliance on compensatory sympathetic activation. Although confirmation in human studies remains limited, Krammer et al. [31] further showed that semaglutide enhanced contractility in human cardiomyocytes by inhibiting late sodium current and reducing SR calcium leak, indicating cardioprotective actions beyond cAMP signaling.

GLP-1 RAs possess substantial cardiometabolic benefits, yet interestingly, clinical and mechanistic evidence indicates no significant effect on suppressing SNS [62]. However, GLP-1 RAs typically cause a modest increase in resting heart rate, attributed primarily to reduced vagal tone rather than direct sympathetic stimulation [63]. Additionally, direct stimulation of GLP-1 receptors within the sinoatrial node can independently accelerate cardiac pacing [61,62]. Although some animal studies suggested potential central sympatholytic effects [64,65], such effects have not translated clearly into reduced sympathetic activity or norepinephrine levels in clinical trials. Therefore, caution is warranted in prescribing GLP-1 RAs, especially for patients with HFrEF, where heart rate control remains crucial [66]. However, in phenotypes such as HFpEF and obesity-related HF, the anti-inflammatory and metabolic benefits of GLP-1 RAs may justify their use, despite the modest increase in heart rate.

The sympathetic nervous system represents a critical yet double-edged sword in HF pathophysiology. Its initial compensatory function to maintain cardiac output evolves into a sustained maladaptive response, causing structural remodeling, biochemical disturbances, and clinical deterioration [38]. Understanding these complex interactions provides not only a deeper mechanistic insight but also potential avenues for targeted therapies aimed at modulating sympathetic activity and improving HF prognosis.

## 4. The Renin–Angiotensin–Aldosterone System in Heart Failure

Central among the maladaptive neurohormonal systems in HF is RAAS, initially serving as a crucial adaptive mechanism to preserve systemic perfusion and maintain homeostasis. However, chronic activation of RAAS promotes pathological alterations that accelerate disease progression and clinical deterioration [67].

**Figure 4:**
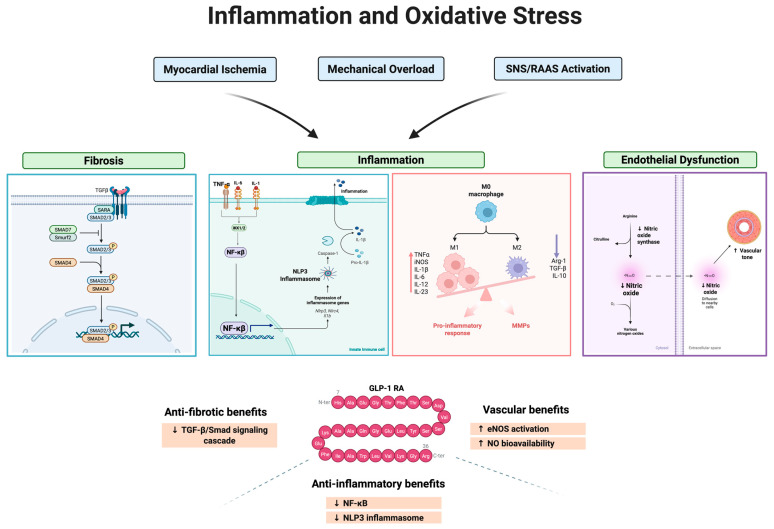
Inflammation and Oxidative Stress Pathways in Heart Failure and Modulatory Role of GLP-1 Receptor Agonists. Arg-1—Arginase-1; eNOS—Endothelial Nitric Oxide Synthase; IL—Interleukin; iNOS—Inducible Nitric Oxide Synthase; MMPs—Matrix Metalloproteinases; NF-κB—Nuclear Factor kappa-light-chain-enhancer of activated B cells; NO—Nitric Oxide; NLRP3—NOD-, LRR- and pyrin domain-containing protein 3; Smad—Small Mothers Against Decapentaplegic; SARA—Smad Anchor for Receptor Activation; TGF-β—Transforming Growth Factor Beta; TNFα—Tumor Necrosis Factor Alpha. Figure created with https://BioRender.com (accessed 4 August 2025).

In the early stages of HF, decreased cardiac output and impaired renal perfusion initiate a compensatory response involving activation of RAAS. Specifically, a decline in renal perfusion pressure, decreased sodium chloride delivery to macula densa cells, and SNS-mediated stimulation of renal juxtaglomerular apparatus (primarily via β1-adrenergic receptors) trigger enhanced secretion of renin, a proteolytic enzyme produced by specialized juxtaglomerular cells in the kidney [68]. Renin cleaves hepatic-derived angiotensinogen into angiotensin I, an inactive precursor. Subsequently, angiotensin I is converted into the potent vasoactive peptide angiotensin II (Ang II) predominantly by angiotensin-converting enzyme (ACE), largely expressed in the pulmonary and systemic vascular endothelial cells [67,68]. Ang II exerts widespread effects through its interactions with angiotensin 1 receptors and causes potent systemic vasoconstriction, significantly increasing total peripheral resistance, resulting in raising cardiac afterload [69]. Initially, this vasoconstriction serves an adaptive role, maintaining adequate systemic arterial pressure and perfusion of vital organs. However, chronic elevation in afterload profoundly impairs myocardial energetics, increasing myocardial oxygen demand and wall stress which drives myocardial hypertrophy, fibrosis, and adverse ventricular remodeling through activation of intracellular signaling cascades, including transforming growth factor-beta (TGF-β), mitogen-activated protein kinase (MAPK), and nuclear factor kappa-B (NF-κB) pathways [70,71]. These molecular cascades not only promote hypertrophic growth and extracellular matrix accumulation but also stimulate inflammatory cytokine production and oxidative stress within cardiac tissue [71] (Figure 1, Figure 2 and Figure 4).

In addition to these direct cardiac and vascular actions, Ang II stimulates aldosterone synthesis and secretion from the adrenal cortex [69]. Aldosterone, a pivotal mineralocorticoid hormone, significantly influences renal sodium and fluid handling. Binding to mineralocorticoid receptors in the renal distal convoluted tubules and collecting ducts, aldosterone enhances sodium reabsorption coupled with potassium and hydrogen ion excretion [68]. Initially, these renal effects increase intravascular volume and cardiac preload, transiently improving cardiac output via the Frank–Starling mechanism. Nevertheless, chronic aldosterone-driven sodium retention contributes profoundly to volume overload, precipitating hallmark symptoms of HF such as peripheral edema, pulmonary congestion, and dyspnea [68,72].

Beyond renal mechanisms, aldosterone exerts direct and robust pro-fibrotic and pro-inflammatory effects on the cardiovascular system by binding to cardiac and vascular mineralocorticoid receptors, and aldosterone induces cardiac fibroblast proliferation, extracellular matrix deposition, and myocardial fibrosis [73,74]. Elevated aldosterone levels correlate with increased cardiac stiffness and impaired ventricular compliance, contributing to the clinical phenotype of diastolic dysfunction, a prominent feature in HFpEF [75]. Furthermore, aldosterone stimulates inflammatory cell infiltration and cytokine production within the myocardium, exacerbating local inflammation, oxidative stress, and further myocardial injury [73,75]. Chronic RAAS activation manifests through distinctive and progressive symptomatology. Patients often present with symptoms indicative of systemic vasoconstriction, such as hypertension and fatigue related to increased afterload and decreased peripheral perfusion. Aldosterone-induced sodium and fluid retention lead to overt clinical manifestations of congestion, including lower extremity edema, pulmonary congestion, orthopnea, and paroxysmal nocturnal dyspnea [76]. Chronic myocardial fibrosis driven by RAAS further impairs ventricular compliance, leading to progressive diastolic dysfunction and worsening symptoms of exertional intolerance and reduced functional capacity [74]. Additionally, aldosterone-induced potassium wasting can cause electrolyte imbalance, predisposing patients to arrhythmias and muscle cramping, further complicating clinical management [77,78].

Studies highlight complex interactions between RAAS and GLP-1 signaling pathways [26,79]. Both systems intersect through regulatory networks implicated in oxidative stress, fibrotic remodeling, and vascular homeostasis. GLP-1 RAs, primarily used in treating diabetes and obesity, exhibit beneficial cardiovascular effects that intersect indirectly with RAAS signaling pathways [80]. These agents have been demonstrated to modestly suppress plasma renin activity, potentially by reducing sympathetic drive or altering renal tubular feedback [81]. Additionally, GLP-1 RAs exert natriuretic effects independent of traditional RAAS inhibition by suppressing renal proximal tubular sodium reabsorption via inhibition of the sodium-hydrogen exchanger isoform 3 [79]. Clinically, this translates into improved fluid homeostasis and reduced preload, complementing conventional RAAS-blocking therapies.

Moreover, GLP-1 RAs possess anti-inflammatory and anti-fibrotic properties, partially counteracting Ang II and aldosterone-induced cardiovascular remodeling [82]. They decrease systemic and tissue-level inflammation through NF-κB inhibition and reduce cardiac fibrosis via suppression of TGF-β and Smad signaling pathways [83,84,85]. Additionally, GLP-1-mediated enhancement of endothelial nitric oxide (NO) bioavailability [86] opposes Ang II-induced endothelial dysfunction, potentially improving vascular compliance and decreasing afterload. Despite these intriguing and beneficial modulatory effects, it is essential to emphasize that GLP-1 RAs do not directly inhibit ACE nor block angiotensin or mineralocorticoid receptors [87]. Consequently, their role remains adjunctive rather than primary therapy within RAAS modulation strategies (Figure 1, Figure 2 and Figure 4).

Chronic RAAS activation substantially influences the development and progression of HF through profound vasoconstrictive, pro-inflammatory, pro-fibrotic, and volume expanding effects. Understanding these complex interactions provides not only deeper mechanistic insights but also therapeutic opportunities aimed at interrupting the pathological neurohormonal cascade characteristic of HF. Emerging data regarding GLP-1 RAs further expand potential avenues for adjunctive HF therapy, underscoring the need for precision-based therapeutic approaches tailored to individual patient pathophysiology.

## 5. Inflammation and Oxidative Stress in Heart Failure

Inflammation in HF originates from diverse triggers, including myocardial injury due to ischemia, mechanical stress from cardiac overload, and prolonged neurohormonal activation involving both the SNS and the RAAS [88]. These stimuli collectively activate innate immune responses, initiating a cascade of inflammatory signaling pathways characterized primarily by increased production and release of pro-inflammatory cytokines. Among these cytokines, tumor necrosis factor-alpha (TNF-α), interleukin-1β (IL-1β), and interleukin-6 (IL-6) play crucial roles [89].

TNF-α, extensively studied in HF, induces cardiomyocyte apoptosis, suppresses myocardial contractility, and promotes pathological cardiac remodeling [90]. Additionally, TNF-α activates nuclear factor-kappa B (NF-κB), a pivotal transcription factor that orchestrates the expression of numerous pro-inflammatory mediators, thus perpetuating inflammation [91]. IL-1β similarly compromises myocardial contractile function and triggers activation of the NOD-like receptor protein 3 (NLRP3) inflammasome, a key intracellular protein complex that further amplifies inflammatory responses [91]. IL-6, frequently elevated in HF patients, stimulates hepatic synthesis of acute-phase proteins such as C-reactive protein (CRP), promotes myocardial fibrosis, and contributes significantly to ventricular hypertrophy and structural remodeling [92,93]. Chronic inflammatory signaling further facilitates recruitment and activation of macrophages within cardiac tissue. Macrophages, particularly those polarized towards a pro-inflammatory (M1) phenotype, exacerbate myocardial injury by releasing additional cytokines, activating matrix metalloproteinases, and contributing to degradation of the extracellular matrix [94] (Figure 4). Persistent macrophage-mediated inflammation significantly reduces myocardial compliance, driving ventricular stiffening and diastolic dysfunction, common hallmarks in HF, particularly in the phenotype with preserved ejection fraction (HFpEF) [95].

GLP-1 RAs exhibit significant anti-inflammatory and anti-fibrotic activities through intricate modulation of intracellular signaling pathways, effectively counteracting cardiovascular remodeling driven by angiotensin II (Ang II) and aldosterone [18]. Upon binding and activation of GLP-1 receptors, one of the key intracellular targets is the nuclear factor kappa B (NF-κB) signaling cascade. NF-κB acts as a master regulator of inflammatory gene transcription, controlling the expression of numerous pro-inflammatory cytokines, chemokines, and adhesion molecules [91] (Figure 4). GLP-1 receptor activation leads to inhibition of NF-κB nuclear translocation and subsequent attenuation of inflammatory signaling, reducing systemic and myocardial inflammation [19]. Through the cAMP-PKA axis, PKA exerts inhibitory effects on NF-κB signaling through several complementary mechanisms that are conserved across many cell types, but are particularly relevant in cardiomyocytes and vascular cells [96,97]. In these contexts, PKA stabilizes inhibitor of κB (IκB), thereby preventing NF-κB dimers from translocating into the nucleus and driving transcription of inflammatory genes [98]. PKA can also directly phosphorylate NF-κB subunits, reducing their DNA-binding activity and transcriptional output [99]. By attenuating NF-κB–mediated pro-inflammatory signaling in cardiomyocytes, endothelial cells, and vascular smooth muscle cells, GLP-1 receptor activation contributes to reduced myocardial inflammation, vascular protection, and limitation of maladaptive cardiac remodeling. Consistent with this framework, Zhang et al. [100] demonstrate that liraglutide suppress NF-κB activation, attenuate downstream inflammatory cascades, and improve vascular homeostasis in models of cardiovascular stress, thereby providing a mechanistic basis for their observed cardioprotective effects.

An increasingly recognized mechanism through which GLP-1 RAs exert their anti-inflammatory effect involves the NLRP3 inflammasome pathway [83]. The NLRP3 inflammasome is an intracellular multiprotein complex critically involved in the initiation and perpetuation of inflammatory responses, particularly through its role in activating interleukin-1β (IL-1β) [101]. Upon sensing various pathological stimuli, such as oxidative stress, mitochondrial dysfunction, and Ang II-induced injury, the NLRP3 inflammasome becomes activated, leading to cleavage and maturation of IL-1β, a highly potent pro-inflammatory cytokine [101,102]. IL-1β, in turn, exacerbates myocardial inflammation, fibrosis, endothelial dysfunction, and adverse ventricular remodeling, slightly contributing to the progression of HF [103]. GLP-1 RAs, through multiple intracellular mechanisms, robustly suppress NLRP3 inflammasome activation [83,104]. By reducing mitochondrial reactive oxygen species (ROS) production and improving mitochondrial function, GLP-1 receptor activation directly decreases NLRP3 activation signals [98,105,106] (Figure 4). This inhibitory effect on NLRP3 subsequently reduces caspase-1 activation, leading to decreased cleavage and release of mature IL-1β. By interrupting the NLRP3-IL-1β signaling axis, GLP-1 RAs modestly reduce myocardial and systemic inflammation, mitigate cardiac fibrosis, and preserve cardiac function and structure [100]. While NLRP3 activation is mechanistically distinct from NF-κB signaling, these pathways are functionally interconnected [107]. NF-κB contributes to the priming phase of NLRP3 activation by upregulating transcription of NLRP3 and pro-IL-1β, whereas the subsequent assembly and activation of the inflammasome complex proceed independently. GLP-1 RAs may modulate both pathways, but emerging evidence highlights direct suppression of NLRP3 as a key contributor to their cardioprotective and anti-inflammatory effects [100].

Furthermore, GLP-1 receptor activation attenuates cardiac fibrosis via inhibition of the transforming TGF-β/Smad signaling cascade. TGF-β is a critical mediator of fibrosis, exerting its effects through phosphorylation and activation of Smad proteins, particularly Smad2 and Smad3 [84]. GLP-1 RAs suppress TGF-β expression and Smad phosphorylation [85,108], thereby reducing extracellular matrix production, collagen deposition, and attenuating fibrosis across multiple tissues, including the myocardium. Additionally, these agents enhance endothelial nitric oxide synthase (eNOS) activation, increasing NO bioavailability [85] (Figure 1 and Figure 4). Increased NO signaling opposes Ang II-induced endothelial dysfunction, reduces oxidative stress, and enhances vascular compliance through cyclic guanosine monophosphate (cGMP)-dependent protein kinase G (PKG) signaling, ultimately reducing afterload and vascular stiffness [109].

These anti-inflammatory, anti-fibrotic, and vascular benefits mediated through the inhibition of NF-κB, suppression of the NLRP3 inflammasome, modulation of TGF-β signaling, and enhancement of NO signaling, underscores GLP-1s significant potential as adjunctive therapeutic agents in managing cardiovascular disease, particularly in HF phenotypes characterized by elevated inflammatory states and metabolic dysfunction. Recognizing inflammation and oxidative stress as integral components of HF pathophysiology has driven exploration of novel therapeutic strategies targeting these mechanisms. GLP-1 RAs, demonstrate notable anti-inflammatory and antioxidant properties, expanding their therapeutic potential into cardiovascular diseases, especially HF. Chronic inflammation and oxidative stress significantly influence HF development, progression, and clinical outcomes. These pathways mediate profound structural and functional myocardial alterations, systemic manifestations, and increased morbidity. Explaining these complex mechanisms underscores the importance of targeted therapies aimed explicitly at inflammation and oxidative stress pathways. Therapeutic agents such as GLP-1 RAs exemplify emerging possibilities in personalized medicine, where comprehensive understanding of pathophysiology guides innovative, patient-tailored therapeutic strategies aiming to improve prognosis and quality of life in HF patients.

## 6. Calcium Handling and Excitation-Contraction Coupling in Heart Failure

Efficient cardiac function relies critically upon the precise coordination of electrical excitation and mechanical contraction [110]. In HF, the complex control of intracellular calcium dynamics is significantly disrupted, which leads to decreased myocardial contractile function, poor diastolic relaxation, increased vulnerability to arrhythmias, and the development of pathological ventricular remodeling [57].

Under physiological conditions, cardiac excitation-contraction coupling initiates with membrane depolarization and opening of voltage-dependent LTCC on cardiomyocyte membranes [53]. The influx of extracellular calcium through LTCC releases calcium from the SR via RyR2 channels, drastically increasing cytosolic calcium concentration [53]. Troponin C is bound by cytosolic calcium, which promotes the development of the actin-myosin cross-bridge and starts cardiac contraction [53]. During diastole, relaxation depends on effective calcium removal from the cytosol. This is primarily achieved by reuptake of calcium into the SR via the sarco/endoplasmic reticulum Ca^2+^-ATPase (SERCA2a) pump, tightly regulated by the phosphoprotein PLB [56] (Figure 3). Additionally, calcium is extruded across the sarcolemmal membrane via the sodium–calcium exchanger (NCX), maintaining calcium homeostasis and cardiac relaxation [111].

In HF, substantial alterations in calcium handling occur at multiple critical sites. A prominent abnormality is reduced SERCA2a expression and function, severely compromising SR calcium reuptake during diastole [58]. Decreased SERCA2a activity delays myocardial relaxation, directly contributing to impaired ventricular filling, diastolic dysfunction, and elevated filling pressures, which manifest clinically as dyspnea and exercise intolerance [58]. Concurrently, PLB remains predominantly unphosphorylated in HF, further suppressing SERCA2a function [58]. Chronic neurohormonal activation, particularly involving persistent sympathetic stimulation, enhances protein kinase A (PKA)-mediated phosphorylation of RyR2 channels, increasing their open probability [54]. Hyperphosphorylated RyR2 channels become leaky, releasing calcium from the SR into the cytosol during diastole. This inappropriate diastolic calcium leak decreases SR calcium stores available for subsequent systolic release, diminishing myocardial contractility and impairing cardiac output. Furthermore, elevated diastolic cytosolic calcium predisposes cardiomyocytes to electrical instability, generating delayed afterdepolarizations and considerably increasing arrhythmogenic potential, contributing to sudden cardiac death in advanced HF patients [54]. Additionally, in failing cardiomyocytes, NCX expression and activity are often upregulated as an adaptive mechanism to extrude excess cytosolic calcium. Paradoxically, increased NCX activity exacerbates intracellular sodium accumulation, disrupting the intracellular sodium–calcium balance and further impairing cellular energetics [111]. Elevated sodium concentrations subsequently increase the workload of the sodium-potassium ATPase pump, compounding myocardial energy demands and precipitating further metabolic dysfunction [111] (Figure 3).

Clinically, disrupted calcium cycling directly correlates with key symptoms and complications observed in HF patients. Reduced SERCA2a function and impaired calcium reuptake clinically manifest as exertional dyspnea, pulmonary congestion, and reduced exercise capacity due to elevated diastolic pressures. RyR2 dysfunction and calcium leakage increase susceptibility to ventricular arrhythmias, significantly impacting prognosis through increased rates of sudden cardiac death and hospitalization [55]. The collective impairment in excitation-contraction coupling also underlies symptoms of fatigue, reduced cardiac reserve, and declining functional status in HF patients.

While GLP-1 RAs are not direct modulators of ion channels or calcium pumps, growing evidence reveals their capacity to indirectly preserve calcium handling integrity and excitation-contraction coupling through interconnected metabolic, oxidative, and anti-inflammatory pathways. In HF, where energetic compromise, oxidative stress, and maladaptive neurohormonal signaling impair calcium cycling, GLP-1 RAs exert cardioprotective effects by stabilizing key nodes of calcium regulation, including SERCA2a and RyR2. One critical site of overlap is the preservation of SERCA2a function. GLP-1 RAs such as liraglutide and semaglutide have been shown to enhance mitochondrial ATP production via AMPK and PGC-1α-mediated pathways, indirectly sustaining the energy-intensive SERCA2a pump activity necessary for diastolic calcium reuptake [112] (Figure 3). Preclinical studies demonstrate that GLP-1 RAs also upregulate the SIRT1/SIRT3 signaling axis [113,114], which activates antioxidant defenses and improves mitochondrial efficiency, alleviating oxidative suppression of SERCA2a activity [59]. Additionally, GLP-1 RAs reduce RyR2 dysfunction by limiting chronic PKA-mediated hyperphosphorylation and ROS-mediated changes [60]. RyR2 stabilization reduces diastolic calcium leak and maintains SR calcium, enhancing contraction and lowering arrhythmia risk [60,115,116]. Animal studies have reported that GLP-1 RAs diminish RyR2-mediated calcium spark frequency and reduce delayed afterdepolarizations, suggesting a mechanistic basis for their observed antiarrhythmic effects in failing myocardium [60].

GLP-1 RAs also intersect with the NO–cGMP–PKG signaling pathway [117,118], which enhances PLB phosphorylation and disinhibits SERCA2a [119]. In ischemia–reperfusion models, semaglutide has been shown to activate the PKG–PKCε–ERK1/2 cascade, attenuating cardiomyocyte apoptosis and improving calcium cycling [34]. By promoting eNOS activity and increasing NO bioavailability, GLP-1 RAs may contribute to downstream enhancement of lusitropic function [120]. Furthermore, GLP-1 RAs exert anti-inflammatory effects by suppressing NF-κB and IL-1β signaling [82,100], known disruptors of calcium homeostasis [121]. The excitation-contraction process is likely kept structurally and functionally intact due to these anti-inflammatory effects. Importantly, GLP-1 RAs mitigate sympathetic overdrive and blunt chronic catecholamine exposure, potentially reducing maladaptive PKA-RyR2 phosphorylation and excessive NCX activation [18,122].

Taken together, GLP-1 RAs influence multiple converging mechanisms that underlie calcium mishandling in HF. By restoring mitochondrial energetics, attenuating oxidative and inflammatory stress, and modulating neurohormonal tone, these agents stabilize SERCA2a and RyR2 function, reduce arrhythmogenic calcium leak, and improve both systolic and diastolic performance. These findings suggest a potential function for GLP-1 RAs as modulators of metabolic-calcium coupling in management of HF. However, clinical caution remains warranted with GLP-1 RAs in HF, particularly in patients with HFrEF due to their modest chronotropic effects, which may exacerbate calcium leakage, arrhythmogenesis, and myocardial oxygen demand. In contrast, for phenotypes like HFpEF, where metabolic dysfunction and inflammation are prominent, GLP-1 RAs may provide net clinical benefits through improvements in metabolic health and vascular function, indirectly supporting more efficient calcium handling and cardiac relaxation.

In summary, disrupted calcium handling and impaired excitation-contraction coupling are fundamental to HF pathogenesis, directly influencing cardiac function, arrhythmia risk, and clinical prognosis. Targeting these pathways remains an active area of therapeutic research, where agents enhancing calcium cycling or reducing associated oxidative stress and inflammation—such as GLP-1 RAs—show promising potential. Ultimately, a deeper mechanistic understanding of calcium handling disruptions in HF is essential to identify precise, personalized therapeutic approaches aimed at improving patient outcomes, clinical stability, and quality of life.

## 7. The Natriuretic Peptide System in Heart Failure

The natriuretic peptide system is an intrinsic, potent cardiovascular protective mechanism that responds dynamically to volume overload, increased wall stress, and hemodynamic imbalance [123]. In the complex pathophysiology of HF, this system functions primarily to counterbalance the damaging effects mediated by sustained neurohormonal activation, especially by the SNS and the RAAS. Despite its initially robust compensatory activation, the natriuretic peptide system eventually becomes functionally impaired during chronic HF [124]. This paradoxical phenomenon, is characterized by elevated circulating levels of natriuretic peptides but diminished biological responsiveness, playing a significant role in the persistent progression of HF.

**Figure 5:**
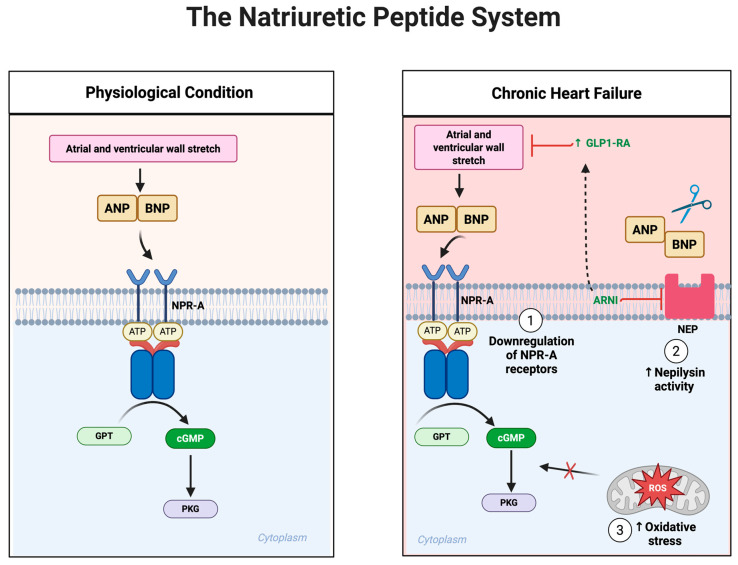
The Natriuretic Peptide System in Physiological and Heart Failure Conditions and Modulatory Role of GLP-1 Receptor Agonists. ANP—Atrial Natriuretic Peptide; ARNI—Angiotensin Receptor–Neprilysin Inhibitor; ATP—Adenosine Triphosphate; BNP—B-type Natriuretic Peptide; cGMP—Cyclic Guanosine Monophosphate; GLP-1 RA—Glucagon-Like Peptide-1 Receptor Agonist; GPT—Guanylate Cyclase–Associated Protein; NEP—Neprilysin; NPR-A—Natriuretic Peptide Receptor A; PKG—Protein Kinase G; ROS—Reactive Oxygen Species. Figure created with https://BioRender.com (accessed 4 August 2025).

Under normal physiological conditions, natriuretic peptides, primarily atrial natriuretic peptide (ANP) and B-type natriuretic peptide (BNP), are synthesized and released from cardiac myocytes in response to increased atrial and ventricular wall stretch, indicative of volume expansion and hemodynamic overload [125]. Upon their release, ANP and BNP exert multiple beneficial actions, mainly through activation of the natriuretic peptide receptor A (NPR-A). This receptor activation induces a conformational change that increases intracellular cGMP, subsequently activating PKG [126] (Figure 5). The PKG-mediated signaling cascade facilitates several critical cardiovascular protective actions, including systemic vasodilation, enhanced natriuresis, increased renal water excretion (diuresis), inhibition of sympathetic tone, suppression of renin and aldosterone release, and significant anti-fibrotic and anti-hypertrophic effects on cardiac tissue [126,127,128]. Mutually, these actions decrease both preload and afterload, reduce myocardial oxygen demand, mitigate myocardial fibrosis, and counteract pathological ventricular remodeling, making the natriuretic peptide system indispensable in maintaining cardiovascular homeostasis. However, as HF progresses to its chronic and advanced stages, a marked reduction occurs in the biological efficacy of natriuretic peptides, despite persistently elevated plasma BNP and NT-proBNP levels [127]. This state of functional resistance or impaired responsiveness develops from several interrelated molecular mechanisms. Chronic exposure to high circulating concentrations of natriuretic peptides leads to downregulation and desensitization of the NPR-A receptors in key target tissues, such as the kidneys and vasculature [129]. This receptor attenuation limits the generation of cGMP, thereby reducing downstream signaling efficiency and diminishing the physiological impact of natriuretic peptides, despite their abundance in circulation.

Another crucial mechanism underlying natriuretic peptide resistance involves enzymatic degradation by neprilysin, a neutral endopeptidase abundantly expressed in renal, pulmonary, and endothelial tissues [130]. Neprilysin rapidly breaks down ANP and BNP, shortening their half-life and limiting bioavailability [130]. In HF, increased neprilysin activity further exacerbates the already compromised signaling cascade. This enzymatic degradation becomes particularly problematic because neprilysin simultaneously targets other beneficial peptides, such as bradykinin and GLP-1, thereby increasing neurohormonal imbalance and reducing overall cardiovascular protective effects [131]. Moreover, the effectiveness of natriuretic peptide signaling can be severely compromised by oxidative stress and inflammation, both prominent features of chronic HF. Reactive oxygen species, generated from dysfunctional mitochondria, NADPH oxidase, and uncoupled nitric oxide synthase, directly interfere with cGMP-PKG signaling pathways [117,125,126]. This oxidative environment can lead to chemical modifications of cGMP or direct nitration and inactivation of PKG, profoundly impairing natriuretic peptide-mediated vasodilatory and anti-fibrotic signaling. Clinically, this failure of the natriuretic peptide system has profound consequences. Persistent fluid retention due to diminished natriuretic response promotes progressive volume overload, evidenced by worsening peripheral edema, pulmonary congestion, ascites, and elevated intracardiac filling pressures [124]. The loss of effective vasodilatory signaling results in sustained systemic vasoconstriction, increased afterload, and hypertension, placing additional stress on the already compromised myocardium. Moreover, the failure of natriuretic peptides to suppress pathological fibrosis and hypertrophy allows unchecked extracellular matrix deposition, myocardial stiffening, and diastolic dysfunction, hallmark pathophysiological features of HF, especially HFpEF.

Although GLP-1 RAs do not directly stimulate natriuretic peptide secretion or interact directly with NPR-A receptors, emerging evidence highlights several indirect and clinically relevant interactions between these pathways. GLP-1 RAs improve glycemic control, reduce adiposity, lower systemic blood pressure, and enhance endothelial function, collectively reducing cardiac wall stress and the pathological stimuli driving excessive natriuretic peptide secretion [52]. Furthermore, GLP-1 RAs potentiate endothelial NO production, enhancing vascular cGMP generation, a pathway that converges downstream with natriuretic peptide signaling. Through such indirect vascular support, GLP-1 RAs may help preserve natriuretic peptide responsiveness despite chronic cardiovascular stress. Another interesting aspect of this interaction arises from the shared degradation pathways involving neprilysin. Both natriuretic peptides and GLP-1 are substrates of neprilysin [132]. Consequently, pharmacological inhibition of neprilysin using angiotensin receptor-neprilysin inhibitors (ARNIs), such as sacubitril-valsartan, simultaneously elevates levels of BNP and GLP-1, potentially creating an additive or synergistic cardioprotective environment [132,133]. This dual elevation could help restore natriuretic peptide efficacy while leveraging the metabolic and endothelial benefits of increased endogenous GLP-1. GLP-1RAs enhance NO bioavailability by increasing eNOS activity through AMPK–Akt pathways, leading to vasodilation, reduced vascular inflammation, and improved endothelial function. Similarly, natriuretic peptides directly induce vasodilation via increased cGMP-dependent signaling, enhancing endothelial function and also indirectly enhancing eNOS-derived NO production [134] (Figure 5). While GLP-1 RAs alone do not directly modulate natriuretic peptide receptor signaling, their ability to improve the cardiometabolic environment may indirectly enhance or maintain the effectiveness of the natriuretic peptide system, particularly in patients characterized by metabolic-driven HF phenotypes.

In conclusion, the natriuretic peptide system serves as a critical intrinsic protective mechanism in cardiovascular regulation, adeptly counterbalancing adverse neurohormonal and hemodynamic stressors in early HF stages. However, chronic disease progression leads to functional impairment, despite persistently elevated circulating peptide levels. The mechanisms behind this impaired responsiveness, including receptor downregulation, enhanced enzymatic degradation, and oxidative-inflammatory disruption of signaling highlight important targets for therapeutic intervention. Emerging therapies, such as GLP-1 RAs and neprilysin inhibitors, offer promising complementary approaches that may indirectly restore or enhance natriuretic peptide responsiveness, supporting cardiovascular function and improving clinical outcomes in chronic HF. Thus, preserving and potentiating natriuretic peptide efficacy through an integrated therapeutic strategy represents a key frontier in personalized HF management.

## 8. Discussion

GLP-1 RAs have emerged as promising therapeutic agents in HF management, particularly in patients with HFpEF and metabolic comorbidities [14,15,66]. Initially developed for glycemic control in type 2 diabetes mellitus, GLP-1 RAs were first approved in the early 2000s, with agents like exenatide and liraglutide demonstrating robust glucose-lowering effects. However, clinical experience and early trials in the 2010s revealed consistent weight loss among patients [18], prompting dedicated studies to evaluate their anti-obesity potential. This culminated in the approval of high-dose liraglutide (3.0 mg) for chronic weight management in 2014 (Europe) and 2015 (FDA) [135,136]. The introduction of semaglutide, which demonstrated unprecedented weight loss (~15% of baseline weight at 68 weeks) in the STEP clinical trial program [137], revolutionized the field, leading to FDA approval of semaglutide 2.4 mg for chronic weight management in 2021 [136]. More recently, the dual glucose-dependent insulinotropic polypeptide (GIP) and GLP-1 receptor agonist, tirzepatide, has shown even greater weight loss (~20%) and received approval for obesity treatment in 2023 [138,139]. These developments have sparked substantial interest in the potential cardiovascular applications of these agents, particularly in HFpEF, a syndrome where obesity is a central phenotypic driver [8,9,14,15]. Despite the high prevalence of obesity in HFpEF [3,4,5,6,7,8,9], no therapies had previously targeted this mechanism directly, creating a compelling rationale for evaluating potent anti-obesity medications in this population. The STEP-HFpEF [14] and SUMMIT [35] trials, which demonstrated that both semaglutide and tirzepatide, respectively, not only induce significant weight loss but also lead to clinically meaningful improvements in HF-related symptoms, physical function, quality of life, and clinical outcomes (tirzepatide), marked a pivotal advance in the treatment of obesity-related HFpEF [14,35].

The mechanistic basis for GLP-1 RA benefits in heart failure involves multiple interconnected pathways that address key pathophysiological processes [18,19,140]. At the cellular level, these agents enhance mitochondrial function through AMPK- and PGC-1α–mediated activation [112], improving ATP production essential for energy-intensive processes like SERCA2a-mediated calcium reuptake during diastole [56,57,58]. This metabolic enhancement is complemented by activation of SIRT1 and SIRT3 signaling [58,113,114], which strengthens antioxidant defenses and stabilizes excitation–contraction coupling. The restoration of efficient calcium handling, particularly through preserved SERCA2a function [57,59] and reduced RyR2-mediated calcium leak [54,60], directly addresses the diastolic dysfunction that characterizes many heart failure phenotypes, especially HFpEF [57,58] (Figure 1 and Figure 3).

Beyond cellular energetics, GLP-1 RAs exert significant anti-inflammatory and anti-fibrotic effects [52,82,83,98] that target the chronic pathological remodeling central to heart failure progression [57,58,71,84]. By inhibiting pro-inflammatory mediators such as NF-κB [91,121] and IL-1β [101,103,104] and suppressing the TGF-β/Smad fibrotic pathway [84,85], these agents help limit structural cardiac damage and improve myocardial compliance. Additionally, their enhancement of endothelial nitric oxide production through AMPK and Akt signaling [34,79,86,118,120] promotes vasodilation, reduces vascular resistance, and improves tissue perfusion, collectively reducing cardiac workload and enhancing exercise capacity [57,58] (Figure 4).

The clinical evidence strongly supports the efficacy of GLP-1 RAs in specific heart failure populations. The STEP-HFpEF trial demonstrated that semaglutide significantly improved symptoms, physical function, and quality of life in patients with HFpEF and obesity, with benefits observed regardless of diabetes status [14]. This finding, reinforced by similar outcomes in diabetic HFpEF patients [15] and the cardiovascular event reduction shown in the SELECT trial [10], suggests that GLP-1 RAs are particularly effective in metabolically driven HF. The obesity–heart failure connection is particularly relevant, as obesity independently contributes to diastolic dysfunction, inflammation, and adverse cardiac remodeling [3,4,5,6,7,8,9,57,58]—all targets of GLP-1 RA therapy. The SUMMIT trial [35] further strengthened this evidence base, demonstrating that tirzepatide significantly reduced the composite endpoint of cardiovascular death or worsening HF events (HR 0.62; 95% CI: 0.41–0.95) and improved health status in patients with HFpEF and obesity over a median follow-up of two years [35]. These consistent findings across different incretin-based therapies reinforce the therapeutic potential of targeting metabolic dysfunction in obesity-related HFpEF.

However, the role of GLP-1 RAs in HFrEF remains less defined. Clinical trials such as FIGHT [12] and LIVE [13] have not demonstrated clear benefits in HFrEF populations, potentially due to lower GLP-1 receptor expression in failing myocardium [17] and the limited direct inotropic effects of these agents. Furthermore, the modest chronotropic effects of GLP-1 RAs, attributed to reduced vagal tone [62,63,65] and direct sinoatrial node stimulation [61,122], raise concerns about their use in patients where heart rate control is crucial, particularly in advanced HFrEF.

The neutral or negative findings of GLP-1 receptor agonists in HFrEF primarily derive from the FIGHT [12] and LIVE [13] trials, both of which had notable methodological and population-related limitations. The FIGHT [12] trial enrolled 300 patients with advanced HFrEF (median LVEF ~25%), the majority of whom were recently hospitalized for acute decompensated heart failure. This population was clinically unstable, older, and highly comorbid, with high background event rates. Within this context, liraglutide did not improve mortality or rehospitalization and raised concerns about potential adverse events, though the trial was underpowered for definitive outcome assessment. Similarly, the LIVE [13] trial included 241 patients with stable chronic HFrEF (LVEF < 45%). While modest improvements in glycemic control and weight were noted, the trial reported increased heart rate and numerically higher cardiac adverse events in the liraglutide arm. Importantly, the study population was heterogeneous, spanning ischemic and nonischemic etiologies as well as both diabetic and non-diabetic patients, further complicating interpretation. The combination of small sample size, short follow-up, advanced disease stage in FIGHT [12], and population heterogeneity in LIVE [13] likely limited the ability of these studies to detect meaningful clinical benefit.

In contrast, the STEP-HFpEF [14,15] and SUMMIT [35] trials demonstrated robust, positive findings in patients with obesity-related HFpEF, populations more closely aligned with the metabolic targets of GLP-1 receptor agonists. STEP-HFpEF [14,15] showed that semaglutide significantly improved symptoms, functional capacity, and quality of life, while SUMMIT [35] extended these findings by demonstrating that tirzepatide not only improved symptoms and biomarkers but also reduced cardiovascular death and HF hospitalizations. These differences likely reflect both stronger trial design and larger study populations, as well as the fact that HFpEF with obesity is a metabolically driven phenotype, where GLP-1 receptor agonists directly target central mechanisms of disease. In summary, while preclinical studies demonstrate myocardial effects of GLP-1 RAs through pathways such as calcium handling and anti-inflammatory signaling, the strongest human evidence indicates that their benefits in HFpEF are primarily mediated indirectly via weight reduction, metabolic improvements, and systemic anti-inflammatory effects.

GLP-1 receptor agonists are not yet part of guideline-directed therapy for heart failure and currently have no FDA-approved indication for heart failure treatment. However, accumulating evidence suggests they may complement established treatments, particularly in obese HFpEF where they target weight, inflammation, and metabolic dysfunction. Unlike SGLT2 inhibitors, which improve outcomes across the HF spectrum [141,142] the benefits of GLP-1 RAs appear confined to this specific phenotype. Their mechanisms also differ from ARNI, which modulates neurohormonal and natriuretic peptide pathways, suggesting potential additive effects. While β-blockers remain central in HFrEF, their role in HFpEF is less clear [141], and the modest heart rate increase associated with GLP-1 RAs warrants caution in patients requiring strict rate control. The dosing regimens used in HF trials (Table 1) followed standard obesity management protocols: semaglutide titrated up to 2.4 mg weekly and tirzepatide up to 15 mg weekly, with gradual dose escalation to minimize gastrointestinal side effects. Should clinicians consider GLP-1 RAs for obese HFpEF patients, they would prescribe these agents for their approved obesity indication, following established obesity dosing guidelines. This use would require careful patient selection, shared decision-making regarding risks and benefits, and close monitoring for both cardiac and gastrointestinal tolerance. GLP-1 RAs may thus serve as an adjunctive therapy in obese HFpEF, complementing SGLT2 inhibitors, ARNI, and lifestyle interventions, while their role in HFrEF remains limited.

Taken together, the neutral outcomes of FIGHT [12] and LIVE [13] should not be interpreted as definitive evidence against the use of GLP-1 receptor agonists in HFrEF, but rather as inconclusive findings limited by trial design and population selection. By contrast, the consistent benefits observed in STEP-HFpEF [14,15] and SUMMIT [35] underscore the therapeutic potential of GLP-1 receptor agonists in obesity-related HFpEF and highlight the need for future trials to explore their role across broader HF phenotypes. The therapeutic potential of GLP-1 RAs may be further enhanced through synergistic interactions with established HF therapies. The shared degradation pathway with natriuretic peptides through neprilysin [131,132] suggests potential benefits when combined with ARNIs, as neprilysin inhibition could simultaneously elevate both BNP and endogenous GLP-1 levels [131]. Additionally, while GLP-1 RAs do not directly inhibit the RAAS [48,68,87], their natriuretic effects [79,81,143] and anti-inflammatory properties [52,82,98] may complement traditional RAAS blockade strategies.

## 9. Conclusions

GLP-1RAs have evolved from glycemic control agents to multifaceted cardiometabolic therapies with growing relevance in HF. Substantial evidence now supports their role in improving cardiovascular outcomes, particularly among patients with obesity-related HFpEF Mechanistically, GLP-1 RAs influence several central pathways in HF pathophysiology, including mitochondrial energetics, calcium handling neurohormonal activation inflammation, fibrosis and vascular function. These pleiotropic effects align with the clinical benefits observed in recent trials.

The STEP-HFpEF and SUMMIT trials represent landmark advances, demonstrating that semaglutide and tirzepatide, respectively, not only produce sustained weight loss but also improve symptoms, physical function, and quality of life in patients with HFpEF and obesity, with tirzepatide also reducing major HF-related clinical events. These findings support a paradigm shift in the treatment of HFpEF, from one-size-fits-all approaches to targeted therapies.

Despite this progress, several unanswered questions remain. The efficacy of GLP-1 RAs in HFrEF is uncertain, and concerns about chronotropic effects warrant caution in patients with advanced systolic dysfunction. Future studies should prioritize defining the patient subgroups most likely to benefit from GLP-1 RA therapy, exploring synergistic effects with existing HF therapies, and investigating the mechanistic underpinnings of their cardiac actions, particularly in HFrEF and non-obese HF phenotypes.

## Figures and Tables

**Table 1:** Clinical Trials of GLP-1 Receptor Agonists in Heart Failure Patients.

Trial Name (Year)	Trial Type	Drug & Dose	Population	Sample Size	Primary Endpoint	Key Results	Follow-Up
STEP-HFpEF (2023) [14]	Randomized, double-blind, placebo-controlled	Semaglutide 2.4 mg weekly SC	HFpEF (LVEF ≥ 45%), BMI ≥ 30 kg/m^2^, No diabetes	N = 529 (263 semaglutide, 266 placebo)	Change in KCCQ-CSS and body weight	Improved HF symptoms, physical limitations, exercise function; Greater weight loss vs. placebo	52 weeks
STEP-HFpEF DM (2024) [15]	Randomized, double- blind, placebo-controlled	Semaglutide 2.4 mg weekly SC	HFpEF, Obesity, Type 2 diabetes	N = 616 (310 semaglutide, 306 placebo)	Change in KCCQ-CSS and body weight	Larger reductions in HF symptoms and physical limitations; Greater weight loss (~6.4%) vs. placebo	52 weeks
SUMMIT (2025) [35]	Randomized, double-blind, placebo-controlled, event-driven	Tirzepatide up to 15 mg weekly SC	HFpEF (LVEF ≥ 50%), BMI ≥ 30 kg/m^2^	N = 731 (364 tirzepatide, 367 placebo)	Composite of CV death or worsening HF events	HR 0.62 (95% CI: 0.41–0.95); Improved KCCQ-CSS and 6MWD	Median 104 weeks
FIGHT (2016) [12]	Randomized, double-blind, placebo-controlled	Liraglutide up to 1.8 mg daily SC	Advanced HFrEF (LVEF ≤ 40%), Recent HF hospitalization	N = 300 (154 liraglutide, 146 placebo)	Global rank score (death, HF rehospitalization, NT-proBNP change)	No benefit demonstrated; Trend toward increased HF hospitalizations	180 days
LIVE (2017) [13]	Randomized, double-blind, placebo-controlled, multicenter	Liraglutide 1.8 mg daily SC	Stable chronic HFrEF (LVEF ≤ 45%) with and without diabetes	N = 241 (122 liraglutide, 119 placebo)	Change in LVEF	No improvement in LVEF; Increased heart rate and serious cardiac adverse events	24 weeks

## Data Availability

Not applicable.

## Abbreviations

The following abbreviations are used in this manuscript:

ACE	Angiotensin-Converting Enzyme
AMPK	AMP-Activated Protein Kinase
ANP	Atrial Natriuretic Peptide
Ang II	Angiotensin II
ARNI	Angiotensin Receptor–Neprilysin Inhibitor
BNP	B-type Natriuretic Peptide
BMI	Body Mass Index
cAMP	Cyclic Adenosine Monophosphate
cGMP	Cyclic Guanosine Monophosphate
CRP	C-Reactive Protein
eNOS	Endothelial Nitric Oxide Synthase
GLP-1 RA	Glucagon-Like Peptide-1 Receptor Agonist
GIP	Glucose Dependent Insulinotropic Polypeptide
HF	Heart Failure
HFpEF	Heart Failure with preserved Ejection Fraction
HFrEF	Heart Failure with reduced Ejection Fraction
IL-1β	Interleukin-1 beta
IL-6	Interleukin-6
LTCC	L-Type Calcium Channel
MAPK	Mitogen-Activated Protein Kinase
NCX	Sodium–Calcium Exchanger
NF-κB	Nuclear Factor kappa-light-chain-enhancer of activated B cells
NLRP3	NOD-like Receptor Pyrin domain-containing 3 inflammasome
NO	Nitric Oxide
NPR-A	Natriuretic Peptide Receptor A
NT-proBNP	N-terminal pro-B-type Natriuretic Peptide
NADPH	Nicotinamide Adenine Dinucleotide Phosphate Hydrogen
PGC-1α	PPAR Gamma Coactivator-1 Alpha
PKA	Protein Kinase A
PKCε	Protein Kinase C epsilon
PKG	Protein Kinase G
PLB	Phospholamban
RAAS	Renin–Angiotensin–Aldosterone System
ROS	Reactive Oxygen Species
RyR2	Ryanodine Receptor 2
SERCA2a	Sarco/Endoplasmic Reticulum Ca2+-ATPase 2a
SIRT1	Sirtuin 1
SIRT3	Sirtuin 3
Smad	SMAD family of signaling proteins
SNS	Sympathetic Nervous System
SR	Sarcoplasmic Reticulum
TGF-β	Transforming Growth Factor-beta
TNF-α	Tumor Necrosis Factor-alpha
